# Long‐Term Psychosocial Outcomes in Japanese Mayer–Rokitansky–Küster–Hauser Syndrome: A Single‐Center Study

**DOI:** 10.1111/jog.70291

**Published:** 2026-04-28

**Authors:** Asuka Okunomiya, Kaori Tsuyuki, Takuma Ohsuga, Tsutomu Ohara, Akihiro Yanai, Rin Mizuno, Koji Yamanoi, Miho Egawa, Masaki Mandai

**Affiliations:** ^1^ Department of Obstetrics and Gynecology, Graduate School of Medicine Kyoto University Kyoto Japan

**Keywords:** infertility, long‐term psychosocial outcomes, MRKH syndrome, sexual function, vaginoplasty

## Abstract

**Aim:**

Vaginal creation enables sexual intercourse in patients with Mayer–Rokitansky–Küster–Hauser (MRKH) syndrome. However, long‐term psychosocial outcomes, particularly in Japanese patients, remain underreported. This study aimed to explore the long‐term psychosocial outcomes associated with different choices of vaginal creation in patients with MRKH syndrome.

**Methods:**

This retrospective study included 24 patients with MRKH syndrome who were managed at Kyoto University Hospital between 1987 and 2024. Medical records were reviewed to evaluate choices regarding vaginal creation, sexual activity, marital status, partner disclosure, reproductive concerns, and psychological well‐being.

**Results:**

Of the 24 patients, 22 had explicitly planned to undergo vaginal creation using surgical methods or nonsurgical Frank dilation, and 19 actually initiated the procedure, although two discontinued it. Among the 17 patients with a formed neovagina, 11 reported sexual intercourse. Seven patients were married, five of whom married partners they met after vaginal creation. One patient delivered via gestational surrogacy. Four patients, including one who declined vaginal creation, developed delayed psychological distress related to femininity and perceived infertility.

**Conclusion:**

Gynecological outpatient care often serves as the primary source of long‐term psychosocial support for patients with MRKH syndrome. Continuous empathetic follow‐up beyond the period of vaginal creation may be essential to address evolving emotional and social challenges.

## 1 Introduction

Mayer–Rokitansky–Küster–Hauser (MRKH) syndrome is a congenital condition characterized by primary amenorrhea and vaginal agenesis resulting from abnormal differentiation of the Müllerian ducts during embryogenesis [1]. Affected individuals have a normal female karyotype (46, XX) and typically present with congenital absence of the vagina and rudimentary bilateral uterine remnants. However, their ovaries and fallopian tubes are anatomically and functionally normal [1].

The primary aim of treatment is to achieve satisfactory sexual function. Vaginal creation, including surgical and nonsurgical approaches, such as the Frank method, is often performed for this purpose [2]. However, it is not strictly essential because patients have normal endocrine function and experience no functional impairment in daily life, apart from limitations in sexual activity and reproductive potential. Several studies have focused on procedural techniques and postcreation sexual function [3, 4], but relatively limited attention has been paid to long‐term psychosocial aspects, such as marriage, reproductive planning, and mental health. A 2009 systematic review, which proposed a cognitive‐behavioral model for psychological distress in MRKH syndrome, reported that only 3% of the literature at the time focused on psychosocial issues [5]. Comprehensive reports from Japan describing social or psychological trajectories after choosing to undergo or decline vaginal creation are lacking. Consequently, a significant knowledge gap persists in relation to the long‐term challenges faced by individuals with MRKH syndrome, the type of care they require, and how gynecological outpatient services can support them beyond procedural management.

To address this issue, we conducted a retrospective study of patients diagnosed with MRKH syndrome at our institution over 30 years to explore the long‐term outcomes in those who underwent vaginal creation and those who did not. We examined their treatment choices, sexual activities, social lives (including marriage), and psychological outcomes.

## 2 Methods

### 2.1 Ethics Statement

This retrospective study used anonymized data extracted from medical records. In accordance with national ethical guidelines for clinical research in Japan, the requirement for written informed consent was waived, but patients were informed through an opt‐out notice on the hospital website. This procedure, along with the study design, was reviewed and approved by the Ethics Committee of Kyoto University Hospital (approval number: R4215).

### 2.2 Study Design and Setting

This descriptive retrospective cohort study was based on a case series of patients with MRKH syndrome who were managed at Kyoto University Hospital between January 1987 and March 2024. We retrospectively followed patients from the time they were referred to our hospital for possible neovaginal creation and evaluated their subsequent sexual, relational, and psychosocial outcomes over time.

### 2.3 Case Identification

Case identification was performed in two phases. We manually reviewed the archived surgical records of patients diagnosed before 2000. For those diagnosed from 2000 onward, we used the structured electronic medical record database of the hospital to search for diagnostic labels such as “vaginal agenesis” and “congenital absence of the uterus.” The diagnosis of MRKH syndrome was confirmed by cross‐referencing clinical summaries, radiological findings, and physician notes.

### 2.4 Data Collection and Interpretation

The following clinical and psychosocial variables were extracted from the medical records: history of neovaginal creation or an explicit plan for it (including both surgical procedures and nonsurgical Frank dilator therapy), history of sexual intercourse, marital status and changes over time, disclosure of MRKH diagnosis to romantic partners, desires or actions related to childbearing, and long‐term psychological status based on physician documentation during outpatient visits or follow‐up consultations. Information regarding reproductive intentions was extracted from physician documentation during routine follow‐up visits. These included documented expressions of interest in biological motherhood, consideration of gestational surrogacy, uterine transplantation, adoption, or oocyte cryopreservation. Reproductive intentions were not assessed using a structured questionnaire; rather, they were identified when patients spontaneously discussed these issues or when such concerns were clinically relevant during follow‐up consultations.

All clinical and psychosocial data were manually extracted from the electronic and archived medical records by a single investigator (A.O.). As psychological findings were derived from physicians' narrative notes rather than from standardized questionnaires, the information was coded descriptively rather than quantitatively. Therefore, this study should be interpreted as an exploratory descriptive analysis rather than a formal psychological assessment. Each case was reviewed to ensure internal consistency across the different follow‐up periods.

Although inter‐rater validation was not conducted owing to the retrospective design and qualitative nature of the data, ambiguous descriptions were discussed by the research team to minimize potential misclassification.

### 2.5 Follow‐Up Protocols

Follow‐up schedules varied depending on the treatment modality and attending physician, but were generally as follows.

For patients undergoing surgical neovaginal creation, frequent visits were scheduled during the first 3 months postoperatively for wound monitoring and management. During the first year, visits typically occurred every 3–4 months. Thereafter, patients were encouraged to attend follow‐ups every 6–12 months to assess the condition of the neovagina and review the evolving needs regarding its use.

For those opting for nonsurgical neovaginal creation (Frank method), monthly follow‐up was recommended during the initial 3 months to ensure procedural stability and allow for gradual up‐sizing of the dilator. Once neovaginal formation was nearly complete, follow‐up intervals were extended to every 3–6 months. When patients chose to suspend Frank therapy due to diminished perceived need, we encouraged biannual visits to maintain supportive contact and facilitate rapid reinitiation of therapy.

The attending physician assessed the neovagina at each follow‐up visit through a pelvic examination, observed the demeanor of the patient, inquired about any recent changes or concerns, and documented any spontaneous remarks made by the patient, except in cases in which Frank dilation therapy was discontinued. Questions regarding the presence of a partner or history of sexual intercourse were not necessarily asked at every visit. Repeatedly raising such topics in the absence of a partner could be distressing for patients and potentially discourage continued follow‐up. In this study, information not documented in the medical records was recorded as “unknown.”

### 2.6 Assessment of Anxiety

“Late‐onset anxiety” was evaluated retrospectively based on physician notes in the medical records. A patient was classified as having anxiety if any of the following criteria were met:

1. Documented consultation with a psychiatrist or another mental health professional;
2. Initiation of anxiolytic or antidepressant medication in any department; or
3. A formal psychiatric diagnosis recorded in the chart.

Transient emotional conflicts, such as hesitation or difficulty disclosing the MRKH diagnosis to romantic partners, were not coded as anxiety. Cases without such documentation were recorded as “no anxiety.”

## 3 Results

### 3.1 Patient Characteristics and Diagnosis

Twenty‐four patients with MRKH syndrome were included in this study (Tables 1 and S1). All patients presented with primary amenorrhea at a mean age of 15 years and were diagnosed with MRKH based on clinical and imaging findings. While one patient (F) had solitary horseshoe kidney suggestive of syndromic (type II or Müllerian duct Agenesis, Renal anomalies, Cervicothoracic Somite dysplasia [MURCS]) MRKH [6], all other patients had isolated MRKH (type I). Decisions regarding neovaginal creation were typically made in late adolescence or early adulthood (18–20 years), although some patients considered treatment later when entering stable relationships.

**TABLE 1:** Summary of key psychosocial outcomes of 24 patients with MRKH syndrome.

Variable	*n* (%)	Comments
Total patients	24	
Type I MRKH (isolated)	23 (96%)	1 type II (MURCS) case
Neovaginal creation	22 planned (92%), 17 completed (71%)	15 surgical, 2 Frank method
Sexual intercourse achieved	11/17 (65%)	Comparable to international reports
Married	7/24 (29%)	5 after neovaginal creation
Partner disclosure (documented)	6/24 (25%)	Gradual/partial in several cases
Late‐onset anxiety	4/24 (17%)	Developed years after creation or diagnosis
Fertility attempts	3/24 (13%)	1 surrogacy, 1 oocyte retrieval, 1 uterine transplant attempt

Abbreviation: MURCS, Müllerian duct agenesis, Renal anomalies, Cervicothoracic Somite dysplasia.

### 3.2 Choices Regarding Vaginal Creation

Of the 24 patients, 22 (92%) had definite plans for neovaginal creation using either surgical or nonsurgical (Frank) methods [7], whereas two (Cases W and X) declined any procedure. Seventeen patients ultimately completed neovaginal formation (15 surgical, 2 Frank), while five discontinued or were transferred before completion. Most surgical procedures were laparoscopy‐assisted Davydov vaginoplasties [8] using peritoneal tissue, with a few earlier cases of McIndoe vaginoplasties [9] using skin grafts. Variations in the vaginoplasty modalities are shown in Table S1.

### 3.3 Sexual Activity

Among the 17 patients who completed the neovaginal formation, 11 (65%) reported engaging in sexual intercourse (Tables 1 and 2). The remaining six patients included four without partners, one who was transferred soon after surgery, and one who developed postoperative stenosis. Pain or bleeding during intercourse was generally mild and transient (Table 2).

**TABLE 2:** Long‐term sexual function and symptoms of patients with MRKH who are sexually active after vaginal creation.

CaseID	Observation period (years)	Time to first intercourse postcreation	Time to last intercourse postcreation	Time point of bleeding during intercourse postcreation	Time point of pain during intercourse postcreation	Time point of perceived shortness during intercourse postcreation
A	11	6 months	11 years	6 months and 10 years	None	10 and 11 years
B	21	Within 10 years	More than 18 years	None	None	None
C	28	3 months	26 years	None	None	None
D	22	1 year	21 years	12, 13, 14, 15, and 20 years	20 years	1, 20, and 21 years
E	3	5 months	10 months	None	None	None
F	1	3 months	10 months	None	10 months	None
G	12	6 years	9 years	None	6 years	None
H	5	5 months	5 years	9 months, 15 months, 4 years, 5 years	None	5 months
I	15	4 years	12 years	None	None	None
J	5	2 years	4 years	None	None	2 and half years
K	5	7 months	5 years	None	None	7 months

Abbreviations: MRKH, Mayer–Rokitansky–Küster–Hauser; ‘none’ indicates no symptoms reported.

### 3.4 Marital Status and Partner Disclosure

Seven patients married during follow‐up, five after neovaginal creation, and one before but without completing the procedure. Most marriages occurred with partners they met after vaginal formation. The patterns of disclosure to romantic partners varied (Table 3). Some patients had already disclosed their diagnosis at referral, while others delayed or partially concealed it even after neovaginal creation. Three patients (H, I, and U) were documented as experiencing gradual or selective disclosure, illustrating the complex interpersonal processes surrounding the communication of the MRKH diagnosis in intimate relationships.

**TABLE 3:** Paraphrased summaries of the experiences of selected patients with MRKH in disclosing their diagnosis to romantic partners based on clinical documentation.

Case	Vaginal creation	Sexual intercourse	Summary
H	Yes	Yes	After vaginal creation, she entered a relationship 2 months later and had her first intercourse 5 months postoperatively. At 9 months, she reported that her partner did not yet know about her condition. At 2 years and 8 months, she married after explaining to him that she could not have children. At 5 years, she transferred to another hospital in hopes of uterus transplantation
I	Yes	Yes	She struggled for years with disclosing her condition and felt anxious about intercourse. At 3 years and 9 months postvaginal creation, she began a relationship with regular sexual activity. By 4 years and 11 months, she had told her partner she could not conceive and felt anxious about meeting his parents. At 5 years and 5 months, she reported good relationships with them. The partner later became seriously ill. At 9 years and 11 months, she began a new relationship but was unsure about disclosing her uterine absence. At 10 years and 5 months, she planned to marry but worried about suspicion due to amenorrhea. At 11 years and 2 months, she married but feared disclosure via insurance paperwork. At 13 years and 2 months, she remained married with good neovaginal condition, though without sexual activity for a year. At 15 years and 1 month, she was asymptomatic and completed follow‐up
U	No	No	She presented for vaginal creation in preparation for marriage and initially requested weight reduction. Three months later, she held a wedding ceremony without registration and reported that she had told her partner about the planned vaginal creation and infertility, but not about the absence of a uterus. At 11 months, she formally registered the marriage after explaining to him the absence of a uterus, infertility, and the need for surgery, framing the condition as acquired for clarity. She self‐discontinued follow‐up at 1 year and 10 months

### 3.5 Reproductive Intentions and Fertility‐Related Experiences

Three patients actively pursued reproductive options (Table S1). One delivered via domestic gestational surrogacy, one underwent oocyte retrieval but could not proceed owing to institutional restrictions, and one was transferred for uterine transplantation.

Two additional women expressed interest in future childbearing: one maintained an open attitude toward multiple options (surrogacy, uterine transplantation, adoption), while another attempted oocyte cryopreservation but discontinued it after experiencing distress about infertility. These findings illustrate the diverse coping patterns for reproductive loss and future planning.

### 3.6 Late‐Onset Anxiety

Four patients developed late‐onset anxiety years after diagnosis or vaginal creation (Table 4). Patient E experienced depressive symptoms 2 years postoperatively, partly linked to amenorrhea‐related self‐consciousness. Patient L developed emotional instability and anxiety about femininity and hormonal issues 9 years after surgery. Patient M began anxiolytic therapy 6 years postoperatively, expressing growing distress regarding infertility and reproductive limitations. Patient X, who declined vaginal creation, reported depression and low self‐esteem at the age of 39 years, which she attributed to congenital vaginal absence. These findings suggest that anxiety can emerge long after initial adaptation and is not limited to surgical experience.

**TABLE 4:** Paraphrased narratives of psychological symptoms developing years after diagnosis or vaginal creation based on clinical documentation.

Case ID	Vaginal creation	Sexual intercourse	Summary
E	Yes	Yes	In the second year postoperatively, she developed depressive symptoms reportedly triggered by work‐related stress and sought psychiatric care. She had a complex about lacking menstruation and avoided contact with others, especially men
L	Yes	Unknown	In the ninth year, she experienced emotional instability and distress about amenorrhea, affecting her sense of femininity. She was also anxious about hormonal issues. Although psychiatric consultation was considered, she declined due to reluctance to disclose her condition
M	Yes	Unknown	Starting in the sixth year, she expressed concern about infertility and began anxiolytics. Although she was advised to seek psychiatric support, she did not. By the fifteenth year, she expressed deep distress regarding uterine transplantation and oocyte cryopreservation, stating that without the prospect of fertility, she could not even consider a partner
X	No	No	Diagnosed in her late teens, she was advised to return for evaluation if she desired vaginal creation, but she declined follow‐up. At age 39, she visited a gynecologist for lower abdominal discomfort and mentioned that her depression and lack of self‐confidence might relate to the absence of a vagina. Vaginal creation was considered but ultimately declined

### 3.7 Patients Who Declined Vaginal Creation

Two patients (W and X) declined neovaginal creation. Patient W discontinued follow‐up, stating that not menstruating was “advantageous,” whereas Patient X reconsidered surgery two decades later but ultimately declined. Both cases demonstrated that patients can live physically untroubled lives without vaginal creation, although anxiety and low self‐esteem may persist or resurface later in life.

## 4 Discussion

### 4.1 Sexual and Relational Outcomes

Most patients in this study achieved satisfactory sexual function after neovaginal creation, which is consistent with previous international reports [10, 11]. This finding indicates that physical outcomes can be favorable when patients receive adequate procedural guidance and long‐term follow‐up, even within Japan's unique sociocultural environment [12, 13, 14]. Importantly, satisfactory sexual function did not necessarily translate into emotional stability or improved self‐esteem, underscoring that psychosocial adaptation extends beyond anatomical success.

### 4.2 Surgical Versus Nonsurgical Approaches

Our observations highlight that long‐term satisfaction is determined less by procedural methods than by individual readiness or psychosocial issues. The Frank method, although technically simple, demands persistence and emotional commitment; discontinuation often reflects emotional fatigue rather than anatomical difficulty. Conversely, surgical approaches may offer faster anatomical results but still require sustained psychosocial assistance. These findings emphasize the central role of patient motivation and continued supportive care rather than the type of procedure itself. Regular follow‐up after either approach provides opportunities to reinforce self‐efficacy and prevent premature disengagement from care.

### 4.3 Sociocultural and Reproductive Challenges Across Life Stages

Women with MRKH syndrome face evolving psychosocial challenges shaped by cultural norms, reproductive expectations, and developmental transitions. Disclosure of the condition within intimate relationships can be emotionally demanding. Even in Western settings, motivations for disclosure often include honesty in serious relationships and allowing partners the opportunity to disengage early [15].

In Japan, where biological parenthood carries strong social value [16] and infertility remains stigmatized [17, 18, 19], disclosure may be even more complex. Societal expectations that couples become “complete” through parenthood [20] can be deeply internalized. Within this context, disclosing MRKH—an absolute infertility diagnosed in adolescence—may represent a substantial psychological burden and contribute to delayed or selective disclosure.

Reproductive expectations constitute an additional and often enduring source of distress. Although neovaginal creation enables sexual intercourse, infertility persists and may resurface as anxiety during life transitions such as partnership formation, marriage, or peers' pregnancies. Previous studies of women with absolute uterine factor infertility, including those with MRKH syndrome undergoing evaluation for uterus transplantation, have reported elevated depressive and somatic traits based on standardized psychological assessments [21]. Similarly, high rates of anxiety and depressive symptoms have been documented across diverse cohorts of women with MRKH [5, 22].

Importantly, recent narrative reviews have emphasized that improvements in anatomical or sexual function do not necessarily translate into sustained psychological recovery, particularly when reproductive aspirations remain unmet [23]. As much of the existing literature derives predominantly from Western populations, culturally contextualized evidence—such as data from Japanese cohorts—is essential. Taken together, these findings indicate that the impact of MRKH syndrome extends beyond diagnosis or surgical intervention and may evolve throughout the life course in response to shifting sociocultural and reproductive expectations.

### 4.4 Clinical Implications: A Lifelong, Multidisciplinary Care Approach

Given these evolving and culturally influenced psychosocial challenges, care for women with MRKH syndrome should adopt a structured, longitudinal, and multidisciplinary approach. Postoperative follow‐up must extend beyond anatomical and sexual outcomes to include ongoing psychosocial assessments, with particular attention to life transitions that may trigger renewed distress. Early culturally sensitive guidance on reproductive options [24], paired with emotionally attuned counseling, may help patients form realistic expectations and reduce later anxiety.

Collaborative care involving gynecologists, mental health professionals, reproductive specialists, and nurses is essential to provide continuity of support that adapts to changing needs over time. Nurses, who often maintain regular and trusted contact with patients, play a pivotal role in identifying subtle emotional changes and facilitating timely referral to psychological services when needed. Establishing coordinated care pathways for long‐term follow‐up can assist patients in navigating disclosure decisions, reproductive concerns, identity formation, and partnership or family planning throughout adulthood. This approach recognizes that the challenges of MRKH syndrome extend beyond surgical success and underscores the importance of comprehensive, lifespan‐oriented support tailored to individual and cultural contexts.

### 4.5 Limitations and Future Directions

This study had several limitations. First, it was a retrospective single‐center study based on a small cohort, which limits its generalizability. Second, psychological data were derived from physicians' narrative notes rather than standardized assessments, introducing potential observer and documentation biases. In addition, reproductive intentions were not systematically assessed using structured instruments but were identified from routine clinical documentation. Therefore, reproductive concerns may have been underreported in patients who did not explicitly raise these issues during follow‐up visits. Nevertheless, the present report represents the first systematic description of the long‐term psychosocial outcomes of MRKH syndrome in Japan. Given the scarcity of such data in East Asian populations, these findings provide a valuable context for understanding how cultural and social factors shape adaptation. Future multicenter and registry‐based studies employing validated psychological instruments are required to confirm and expand these observations.

In summary, the experience of women with MRKH syndrome extends far beyond surgical reconstruction. While most patients achieve satisfactory anatomical and functional results, long‐term psychosocial adaptation varies considerably and may involve delayed anxiety or reproductive grief. Therefore, continuous empathetic follow‐up and interprofessional collaboration are essential for promoting lifelong well‐being in this unique population.

## Author Contributions

A.O. and K.Y. conceived the study and contributed to the study design and data analysis. A.O. extracted the data. A.O. and A.Y. contributed to data collection and organization. A.O. drafted the manuscript. K.T., K.Y., and M.E. critically revised the manuscript. M.M. supervised the study. R.M., T. Oha., T. Ohs., and M.M. provided clinical care for the patients. All authors reviewed and approved the final version of the manuscript.

## Funding

This research received no specific grant from any funding agency in the public, commercial, or not‐for‐profit sectors.

## Disclosure

The authors have nothing to report.

## Ethics Statement

This study was approved by the Ethics Committee of Kyoto University Hospital (approval number: R4215). In accordance with national ethical guidelines for clinical research in Japan, the requirement for written informed consent was waived, and patients were informed via an opt‐out notice on the hospital website.

## Consent

No written informed consent was obtained because only de‐identified clinical information was used, and the Ethics Committee approved an opt‐out approach.

## Conflicts of Interest

Dr. Miho Egawa is an Editorial Board member of *JOGR* and a co‐author of this article. To minimize bias, they were excluded from all editorial decisions related to the acceptance of this article for publication.

## Supporting information


**Table S1:** Clinical and psychosocial characteristics of patients with MRKH syndrome.

## Data Availability

The datasets analyzed in this study contain highly sensitive clinical information and detailed psychological narratives derived from medical records of a very small and potentially identifiable patient population with Mayer–Rokitansky–Küster–Hauser syndrome. Because these data cannot be fully anonymized without risking re‐identification, public sharing of the raw datasets is not permitted under the conditions of the institutional ethics approval (Kyoto University Hospital Ethics Committee, approval no. R4215) and the national “Ethical Guidelines for Life Science and Medical Research Involving Human Subjects” in Japan. De‐identified aggregated data supporting the findings of this study may be made available from the corresponding author upon reasonable request and pending additional institutional approval. No public repository deposit is possible due to these privacy and ethical restrictions.
