# Supplementary material for: Long‐Term Psychosocial Outcomes in Japanese Mayer–Rokitansky–Küster–Hauser Syndrome: A Single‐Center Study
Source: J Obstet Gynaecol Res. 2026 Apr 28;52(5):e70291. doi: 10.1111/jog.70291 (PMC13124316; doi:10.1111/jog.70291)
Supplement: Supplementary file 1 — Table S1: Clinical and psychosocial characteristics of patients with MRKH syndrome. [file JOG-52-0-s001.docx]

# Supplementary Table 1. Clinical and psychosocial characteristics of patients with MRKH syndrome

| Case ID | Age at referral for neovaginal creation | Vaginal creation modalities | Observation period (years) | Sexual intercourse after vaginal creation | Marriage | Status of notification to partners | Desire to have a child | Late-onset anxiety after vaginal creation |
| --- | --- | --- | --- | --- | --- | --- | --- | --- |
| A | 19 | McIndoe | 11 | Yes | No | Unknown | Unknown | No |
| B | 19 | McIndoe | 21 | Yes | Yes | Unknown | Unknown | No |
| C | 23 | Davydov | 28 | Yes | Yes | Already disclosed at referral | Surrogate motherhood | No |
| D | 27 | Davydov | 22 | Yes | Yes | Unknown | Unknown | No |
| E | 18 | Davydov | 3 | Yes | No | Unknown | Unknown | Yes |
| F | 19 | Davydov | 1 | Yes | No | Already disclosed at referral | Unknown | No |
| G | 22 | Davydov | 12 | Yes | Yes | Unknown | Oocyte retrieval only | No |
| H | 24 | Davydov | 5 | Yes | Yes | Gradual disclosure | Transferred seeking uterus transplant | No |
| I | 29 | Davydov | 15 | Yes | Yes | Gradual disclosure | Unknown | No |
| J | 21 | Frank | 5 | Yes | No | Unknown | Unknown | No |
| K | 17 | Frank | 6 | Yes | Being considered | Already disclosed at referral | Always in mind | No |
| L | 19 | McIndoe | 9 | Unknown | No | Unknown | Unknown | Yes |
| M | 20 | Davydov | 19 | Unknown | No | Unknown | Concerns about egg freezing | Yes |
| N | 29 | Davydov | 11 | Unknown | No | Unknown | Unknown | No |
| O | 30 | Davydov | 2 | Unknown | No | Unknown | Unknown | No |
| P | 20 | Davydov | 2 | Postoperative restenosis | No | Unknown | Unknown | No |
| Q | 20 | Davydov | 0 | Transferred to another hospital right after surgery | No | Unknown | Unknown | No |
| R | 16 | Frank | 5 | Vaginal creation interrupted | No | Unknown | Unknown | No |
| S | 21 | Frank | 0 | Vaginal creation interrupted | No | Unknown | Unknown | No |
| T | 19 | Planned for surgery, but transferred | 0 | Planned for surgery, but transferred | No | Unknown | Unknown | No |
| U | 27 | Planned for surgery, but not done | 2 | No vaginal creation | Yes | Gradual disclosure | Unknown | No |
| V | 24 | Planned for surgery, but not done | 0 | No vaginal creation | No | Unknown | Unknown | No |
| W | 16 | No desire for vaginal creation | 2 | No vaginal creation | No | Unknown | Stated that she preferred to avoid marriage | No |
| X | 18 | No desire for vaginal creation | 21 | No vaginal creation | No | Unknown | Unknown | Yes |

Abbreviations: MRKH, Mayer-Rokitansky-Küster-Hauser
